# How Do We Keep our Heads above Water? An Embedded Mixed-Methods Study Exploring Implementation of a Workplace Reintegration Program for Nurses Affected by Operational Stress Injury

**DOI:** 10.3390/ijerph20116037

**Published:** 2023-06-02

**Authors:** Chelsea Jones, Elly O’Greysik, Brenda Juby, Shaylee Spencer, Michelle Vincent, Lorraine Smith-MacDonald, Colleen Mooney, Suzette Brémault-Phillips

**Affiliations:** 1 Heroes in Mind, Advocacy and Research Consortium (HiMARC), Faculty of Rehabilitation, University of Alberta, Edmonton, AB T6G 2G4, Canadasuzette2@ualberta.ca (S.B.-P.); 2 Alberta Health Services, Edmonton, AB T5J 3E4, Canada; 3 Faculty of Nursing, MacEwan University, Edmonton, AB T5J 4S2, Canada; 4 St. Stephen’s College, University of Alberta, Edmonton, AB T6G 2J6, Canada; 5 Edmonton Police Service, Edmonton, AB T5H 0H7, Canada; 6 Department of Occupational Therapy, Faculty of Rehabilitation Medicine, University of Alberta, Edmonton, AB T6G 2G4, Canada

**Keywords:** return-to-work, workplace reintegration, nursing, workplace health and safety, operational stress injury, post-traumatic stress injury, trauma, mental health, post-traumatic stress disorder, workplace injury

## Abstract

Background: Nurses are exposed to potentially psychologically traumatic events which can lead to operational stress injuries (OSI). Workplace reintegration after an OSI can be challenging, especially with repeated exposure to potentially traumatic scenarios and workplace demands. A workplace reintegration program (RP) originally developed for police officers may be of benefit for nurses returning to work after an OSI. The purpose of this study is to investigate the perceived need for an RP for nurses, and its potential contextualization and implementation in the nursing context using an implementation science approach. Methods: This mixed-methods study collected data via questionnaires and focus groups from acute care nurses in Canada (*N* = 19). Data analysis was conducted using descriptive statistics, thematic analysis, and an organizational readiness assessment. Results: Study participants indicated that formalized processes were rarely used to support nurses returning to work after time off due to mental health challenges. Themes included (1) “The Perfect Storm”: the current state of return-to-work, (2) Integral Needs, and (3) A Break in the Clouds: hope for health. Conclusions: Exploration of innovative programs such as the RP may provide additional support to nurses affected by OSIs. Further research is needed regarding workplace reintegration for nurses, and contextualization and evaluation of the RP.

## 1. Introduction

### 1.1. Background

Nurses who are regularly engaged in fast-paced, high-stress environments may be subjected to considerable psychosocial stressors [1]. Compared to the general population, nurses experience much higher rates of mental health conditions, including post-traumatic stress disorder (PTSD) [2]. While the COVID-19 pandemic exacerbated this [3], moral distress and operational stress injuries (OSI) were affecting nurses prior to this global event [2]. OSIs include a broad range of conditions, including PTSD, depression, anxiety, concurrent mental health disorders, and moral injury [4], which interfere with daily functioning [5]. This can result in absenteeism from work, and in some cases, nurses choose to leave the profession altogether. The Alberta Workers’ Compensation Board acknowledges that “psychological injuries are unique and can often be very complex (…) early intervention is key to achieving successful return-to-work outcomes and lessening the impact of work-related psychological injuries” [6].

The current state of the literature regarding return-to-work and workplace reintegration has largely focused on military and public safety personnel (PSP). Presently, in Canada, the term PSP encompasses personnel who ensure the safety and security of Canadians (e.g., border services officers, correctional workers, firefighters, paramedics, police, search and rescue personnel, etc. [5]). Despite the recognition of workplace reintegration as a challenge among this population, there is a paucity of literature that focuses on return-to-work or workplace reintegration for nurses and healthcare professionals. A recent scoping review found limited peer-reviewed literature that addressed accommodations, policies, and programs around return-to-work for nurses, following absences due to mental health challenges [7]. It is also evident that most of the literature regarding workplace reintegration of nurses after mental health challenges is specific to the American and military nursing context. The literature regarding workplace reintegration policies, procedures, and programs for PSP with an OSI is evolving and becoming slightly more prevalent.

Among PSP, there exist a few initiatives addressing the workplace reintegration gap in recovery after an OSI. One such initiative is the Edmonton Police Service (EPS) Reintegration Program (RP). In 2009, the EPS in Canada identified a need to assist police officers who were off work following a critical incident (e.g., officers involved in a shooting) and who had received clinical interventions for mental health concerns. In response, the EPS formalized their workplace reintegration efforts by providing officers with peer support to process their critical incident and regain confidence with operational skills. Over time, the EPS RP evolved and has spread to multiple other PSP organizations across Canada, New Zealand, and the United States. Although RPs may include elements specific to each PSP organization and profession, the goal of the programs remains to assist PSP to return to work as soon as possible following a critical incident, illness, or injury, while diminishing the potential of long-term psychological injury [8].

The RP incorporates both a short- and long-term critical incident RP stream [8]. Detailed descriptions of the RP have previously been published [9]. Inclusive of relationship-building, reintroduction to equipment, skill-building, and exposure to operational scenarios, the participating PSP is guided by a trained peer RP facilitator through a step-by-step process that addresses the unique stressors that the PSP may experience [8]. The support offered by the RPs is complementary to clinical interventions but is outside the scope of what officers receive from their healthcare professionals (i.e., psychologists, occupational therapists, etc.) [8]. The RPs may assist workers’ compensation organizations to facilitate positive outcomes for officers attempting reintegration after developing an OSI. To date, information regarding the potential success of the program has been largely anecdotal. There has been only preliminary research regarding the effect of the RP Facilitator Training Program developed by EPS on the mental health knowledge, attitudes [10], and perspectives of attendees and stakeholders [11,12].

Sustainable, usable, context-specific, and evidence-based options for facilitating workplace reintegration after an OSI are needed to bridge the gap between clinical interventions and the workplace, not only for PSP, but for nurses and other healthcare professionals. Existing programs such as the RP may hold promise for PSP as a starting point for the adaptation or complete development of an approach to workplace reintegration for nurses. Prior to modeling a possible approach using an existing one from a different context, a methodical and guided approach that emphasizes implementation science is advisable to assure the proposed innovation warrants the investment of resources.

### 1.2. Purpose

The purpose of this embedded mixed-methods study was to explore the perceived need as well as potential adaptation, contextualization, and implementation of the RP for emergency room (ER) and intensive/critical care unit (ICU/CCU) nurses in urban centers, using an implementation science approach.

## 2. Materials and Methods

### 2.1. Study Design

This study utilized an embedded mixed-methods study design [QUAL quan] as a component of a wider implementation science framework guided by the Active Implementation Frameworks (AIFs; [13]). Implementation is defined as a specific set of activities designed to put into practice an activity, program, practice, or policy [13,14]. Implementation science refers to the study of the activities, such as methods and strategies, used to facilitate the uptake of programs, practices, and policies [14]. This approach allows for pragmatic and actionable results for the organization, considering implementation of a prospective initiative in combination with more traditional research methods.

**Figure 1:**
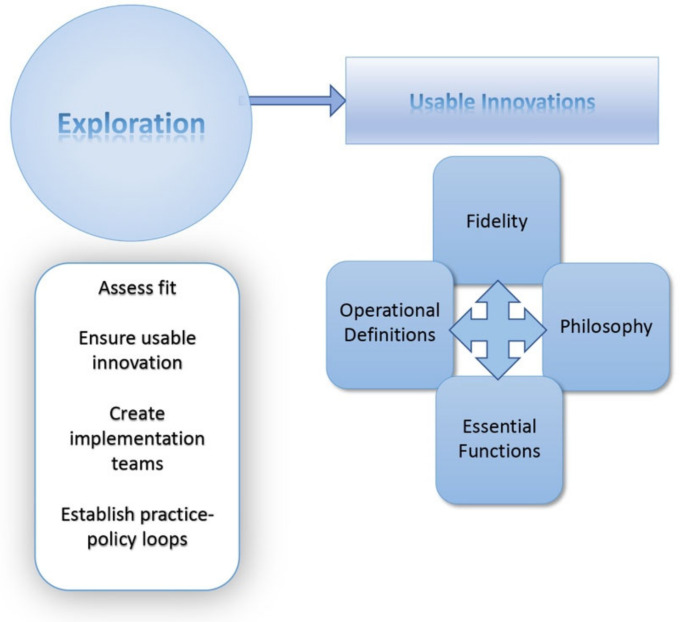
Active Implementation Frameworks: Usable Innovations [13].

**Figure 2:**
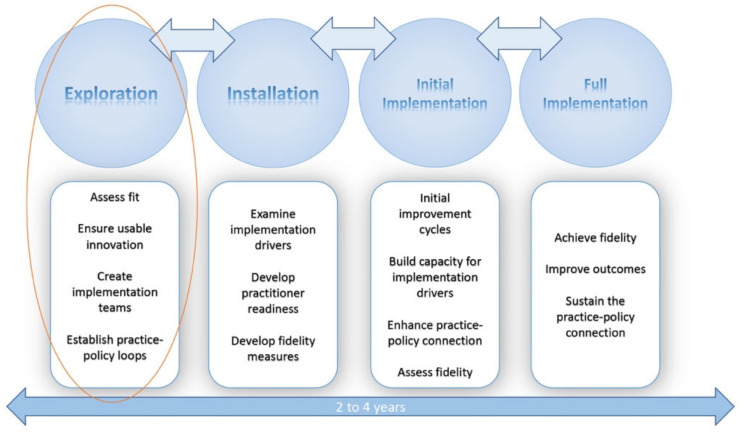
Active Implementation Frameworks’ phases [13].

The AIFs are a set of implementation frameworks that map an integrated approach to implementation practice, science, and policy [13]. The AIFs consists of five core components: (1) Usable Innovation, (2) Implementation Drivers, (3) Implementation Stages, (4) Improvement Cycles, and (5) Implementation Teams [13]. This study pertains to the exploration of an initiative that has not yet been installed and implemented. As such, the frameworks encompassing the implementation research will be limited to the Usable Innovations (Figure 1) and Implementation Stages (Figure 1 and Figure 2) frameworks.

The process was also informed by stakeholder and community-engaged research practices [15,16].

### 2.2. Participants and Recruitment

The study participants included Registered Nurses, Registered Psychiatric Nurses, Licensed Practical Nurses, or Nurse Practitioners working within the ER or ICU/CCU in urban centers in Alberta, Canada. The study participants belonged to provincial health organizations. Participants were recruited through social media websites, such as Twitter and Facebook, as well as through purposeful sampling. Key contacts within nursing organizations, such as peer support groups, assisted with dissemination of study flyers throughout their networks. The care managers of the targeted units were also asked to spread recruitment emails widely, and to post hardcopy flyers in areas frequented by potential participants. Potential participants were asked to contact the research team directly and were screened for inclusion before consenting to study participation.

### 2.3. Data Collection

Data were collected between June and December 2022. Consent and demographic questionnaires were administered via REDCap (Research Electronic Data Capture), which is a secure, web-based software platform [17]. Questions regarding exposure to traumatic events, mental health utilization, and work reintegration were also included in these questionnaires. A semi-structured focus group script, based on the AIFs [13], was designed by the research team to deductively approach data collection. The semi-structured interview script aimed to gather information based on the Exploration Phase of the AIFs (Figure 1) and the Usable Innovations Framework, which include assessments of fit and usability (Figure 1).

Organizational readiness for implementation of the RP for ER and ICU/CCU nurses was also explored, with data capturing of readiness conducted using the Hexagon Tool. This implementation science tool is commonly utilized by implementation researchers to help explore and organize prospective and existing programs and practices [18]. It successfully facilitates discussion and consideration of six key contextual factors that are important to consider prior to trialing new initiatives [18]. These include Need, Support, Capacity, Fit, Usability and Evidence [18]. This tool guides implementation researchers through a series of key questions in each of the six domains and provides criteria to assign a numeric value on a one-to-five scale. The higher the score, the more ready the organization and context is to implement the initiative [18]. Within the context of the AIFs, the Hexagon Tool can be used in the Usable Innovations and Exploration Stage of the AFIs when a site or organization is considering implementation of new programs or practices [18].

The research team applied this tool to the long-term RP to gather information about four specific program indicators: Need, Support, Capacity, and potential Fit. Two further program indicators, Usability and Evidence, were assessed by the research team separately through the team’s knowledge of the current state of the RP program in the PSP context, as well as a scoping literature review.

Qualitative data were captured over eight 60 min focus groups, conducted, and recorded via videoconferencing with Zoom, according to the existing literature and guidelines [19]. Research team training ensured fidelity and consistency across focus groups. During focus groups, the participants were shown a video by a Canadian municipal police force introducing the long-term RP. Questions concerned the potential need for an RP for nursing populations, and how an RP could be contextualized to this population. Data collection continued until information power was reached based on the process outlined [20]. Information power considers the study aim, sample specificity, established theory, quality of dialogue, and analysis strategy, which would ideally also correlate with data saturation.

### 2.4. Data Analysis

Quantitative data were analyzed descriptively using Microsoft Excel software (Microsoft Corporation, USA). Audio and video-recorded focus groups and interviews were thematically analyzed following an iterative inductive and deductive process [21]. Initial codes were developed by identifying themes that emerged from the data. Three researchers independently conducted open coding for each focus group, after which an arm’s-length researcher reviewed and provided feedback on the preliminary themes and codes. Analysis of the preliminary themes followed, including discussion around any conflicting ideas. Disagreements were addressed in a group meeting that involved the arm’s-length senior researcher with expertise, and thematic analysis. Once the final themes were determined, key quotes that illustrated the themes were isolated, and the final presentation of the thematic analysis was prepared.

Additionally, the information gathered through the questionnaires and focus groups allowed the research team to triangulate the data and explore organizational readiness to potentially implement an RP. The Hexagon Tool (University of North Carolina, Chapel Hill, USA) helped to identify program indicators, facilitators, and barriers that may need to be addressed and explored further if the long-term RP were to be implemented within the current nursing context.

## 3. Results

### 3.1. Quantitative: Questionnaire

Demographic details of study participants (*N* = 19) are presented in Table 1. Notably, all participants stated they had been exposed to traumatic events. The vast majority reported this caused some psychological distress (89%). Further, many participants (63%) also stated that they sought mental healthcare for this, and 42% required time away from work as a result. Very few (11%) of those who required time away from work were engaged in a return-to-work or workplace reintegration process. The average age of participants was 36.5 years of age (*SD* = 6.68). The average number of years participants were in the profession of nursing was 12.4 (*SD* = 5.83), with a mean of 10.2 within the provincial health organization *(SD* = 5.05).

### 3.2. Qualitative: Thematic Analysis

**Figure 3:**
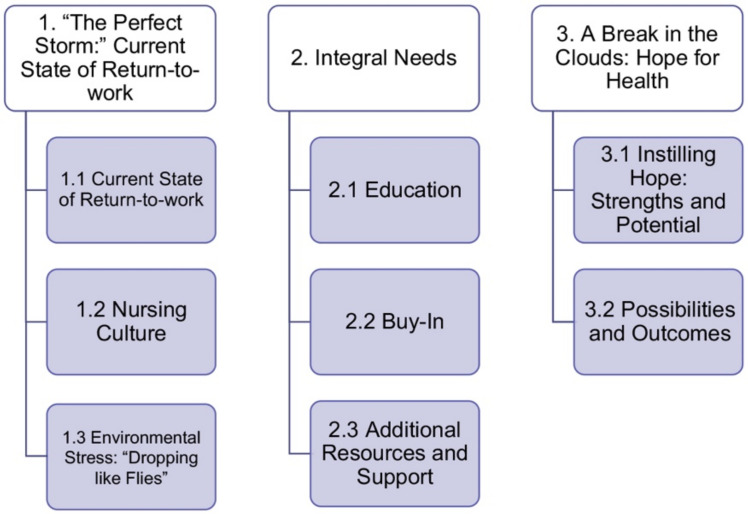
Overview of thematic analysis results.

Thematic analysis of focus group data from participants (*n* = 12) yielded three main themes: (1) “The Perfect Storm”: the current state of return-to-work, (2) Integral Needs for an RP, and (3) A Break in the Clouds: hope for health (Figure 3). Despite approaching the focus groups with a more deductive data collection approach, it was observed through inductive analysis that the participants wished to share insight on topics aside from the RP, which were reflective of ongoing challenges for the nursing profession. Despite the rich discussions not always being specific to the RP, the challenges they identified would directly or indirectly affect the possibility of implementation and adoption of the RP within their context. The qualitative data exhibited dense sample specificity and strong, focused dialogue which allowed the perceived information power to be reached with very few participants [19]. Saturation was reached after six participants. No new patterns emerged thereafter. The themes are summarized below, with detailed descriptions and supporting quotes.

#### 3.2.1. “The Perfect Storm”: The Current State of Return-to-Work

Participants described current workplace realities, as well as how return-to-work was addressed in their workplaces for individuals reintegrating after a psychological injury. Some of the participants shared their experiences or recounted what they had witnessed when other nurses attempted to return to their pre-injury roles. The participants noted multiple challenges in the current approach to return-to-work within their organization. Although they recognized a need for a formalized RP for nurses, they had difficulty envisioning its implementation, based on a stressful environment with a number of barriers that were difficult to overlook.

##### Current Approach to Reintegration and Return-to-Work

Participants overwhelmingly expressed that the current system for return-to-work after an OSI was “broken”, or a short-term “band-aid fix.” They reported that debriefing after a critical incident, which had previously been identified as being helpful to some of the participants, had recently ceased due to increased demands on the healthcare system. The participants perceived that units within the larger organization were inconsistent with their return-to-work approach, and workplace reintegration efforts lacked direction, flexibility and employee input. They expressed that the approaches lacked trauma sensitivity, with a deficiency of recognition of scenes and situations within the work environment that elicit a heightened emotional response. While many participants acknowledged the existence of an employee assistance program offered by the employer, they expressed that engaging with this had been cumbersome, unhelpful and, at times, retraumatizing. Another barrier recognized by the participants was the lack of presumptive legislation for psychological injury for the nursing profession in their jurisdiction, which limited their access to OSI rehabilitation programs through the workers’ compensation organization. Presumptive legislation accepts disease or disorder claims from workers, such as PSP, without the worker having to prove that the physical or psychological disease or disorder resulted from the job [21].

*“You are re-traumatized every time you have to explain yourself. Every time you have to justify why you are off and every time you have to explain what happened… we have to make that a little bit simpler, a little bit easier on the people who are utilizing it [employee assistance program]. And if it’s through our employer, they do have a mandate to protect their employees.”*

*FG 5*

*“There is no presumptive PTSD legislation for nurses here. That is huge. Why would I work someplace where if I got injured, they’ll just throw me to the wolves, as opposed to I could work in another province, make a little less but if I get injured, I’m taken care of.”*

*FG 8*

##### Nursing Culture

Participants reflected both on their love for the nursing profession and pride in their work. In addition, participants expanded on aspects of nursing culture that make recovery from mental health challenging and returning to the workplace problematic. Of note was the increased pressure they put on themselves and expectations they had of others. Participants expressed concern that nurses can be very judgemental and hard on each other. They also reported that these elements of nursing culture have made it difficult to think positively about the trajectory of their career and have interfered with their ability to care for patients and colleagues.

*“We have been pushed for so long, so hard. It’s hard for others to have compassion like well, why are you getting extra special treatment, right?”*

*FG 8*

*“People are constantly judging and talking, and making opinions about different nurses and coworkers. Even when you hear, you know, ‘oh so and so is on stress leave or sick leave or I have not seen so and so, oh they are doing this again’. So I don’t know, it’s, it’s a lot of (laughs) it can be a lot of cattiness. A lot of judgments. I don’t know. Unnecessary judgment on the unit and opinions.”*

*FG 2*

##### Environmental Stress: “Dropping like Flies”

All participants iterated that they were working in environments of extreme stress. An overarching theme of being asked to do more with less by the unit, hospital, organization, and government was emphasized. A nation-wide nursing shortage was noted to be at the root of the taxing environment. The participants spoke of pressure to pick up extra shifts and overtime, and identified an overwhelming guilt if they could not do so. The invasiveness of the demand for staffing included constant phone calls and text messages requesting them to work, working short-staffed shifts, missing breaks, and not having adequate coverage to address the needs of their patients with integrity and safety. Distress among participants was evident as they described questionable quality of care being provided to patients. The potential impact both on patient safety and on the physical and psychological safety of themselves and their peers was concerning to them. Participants also conveyed an overarching mistrust of other stakeholders that extended to all levels of the organization.

*“…but if the building was on fire they would have a duty to provide a safe working environment and they did not do that…we were dropping like flies, we were all being burned to death. And now we are seeing the results of that, and the supports are not sufficient to get us back. So, this is what this reintegration program is necessary for.”*

*FG 5*

*“…we have had two suicides (crying). We have lost people to that…it’s a big wakeup call…I worry about my colleagues. The pandemic has been, like I said exemplified and brought out stressors that…I don’t know…it has just made the cracks more visible.”*

*FG 3*

*“Not the way you feel like any kind of, dignified person or dignified care would be provided to these people [the patients]… I think it’s just going to get worse. They [new staff] are learning a different unit because everyone is tired so they are not being taught what you think would be appropriate ICU care…everyone is just trying to keep their head above water.”*

*FG 2*

#### 3.2.2. Integral Needs of an RP

Nurse participants identified that a formalized RP was desired. They also struggled to envision how this could be implemented within their current work environment. Despite these challenges, they were able to highlight some integral needs for implementation of an RP, with emphasis on education, buy-in, and the need for resources and support.

##### Education

Participants noted the need for meaningful education, organized by the employer, related to mental health and wellness, trauma, symptoms of OSIs, and self-care. They envisioned this as a yearly certification program or something more substantive than resources posted on the intranet or emailed to staff. Participants reflected that modules through the employee education system were not considered meaningful but allowed the organization to “check boxes” that they were supplying mental health education. Adequate education would allow nurses to know about the RP and know when they may need to engage in this service or other mental health resources.

*“Resiliency, education, psychoeducation, tactics, mindfulness techniques, really anything that supports mental health.”*

*FG 8*

*“If they are making us do ‘It’s Your Move’ (education for safe client handling) for your back, they need to do something for mental health.”*

*FG 5*

*“…not sending out an email with a picture and a video of ‘watch this,’ it’s not going to happen. People need actual education… I think the culture would start shifting on its own and allowing people saying it’s okay for you to call in sick because you are mentally not okay.”*

*FG 8*

##### Buy-In

Widespread buy-in was noted by participants to be paramount for the implementation and sustainability of the RP. This was emphasized at the organizational and management level, with a desire for the RP to utilize a top-down approach to implementation. With a bottom-up approach at the unit level, participants feared the program would not succeed due to a lack of resources and support, including staff shortages and inconsistencies in policies and procedures. It was stressed by participants that buy-in and faith in the RP would be needed at all levels of the organization, from front-line to administrative staff. They also felt the widespread public perception was that OSI happened to PSP, but not nurses. Societal buy-in and understanding that other professions can experience workplace trauma and OSI were also discussed as being needed.

*“It would take a true committed investment from (an organizational) standpoint. In both recognizing the importance of investing in their staff, as well as, investing staff in their staff.”*

*FG 3*

*“Unless you have the buy in you are not going to have the cooperation…unless you prove that there is value, you are not going to have that buy in.”*

*FG 3*

##### Additional Resources and Support

Participants identified additional needs that would facilitate work reintegration. These included adequate financial support and physical space to allow for a separate reintegration unit. Further, participants identified the need for appropriately trained supernumerary staff who would be involved in the RP to ensure they understand the current environment and skill set of the nurse reintegrating. Employing personnel that are “*the right fit*”, for a reintegration team was noted as important to the potential success of the program. Flexibility in organizing return-to-work processes was also reported by participants as an essential component of supporting staff due to shift work. Nurse participants reflected on the importance of peer support and mentorship both within and outside the RP.

*“…that reintegration piece, having someone supportive with you, and you’re able to show up, and if you’re distressed, you’re able to leave, that ability to leave when you are feeling unsafe…Myself and a reintegration person could go into the emerg with my scrubs on and watch a code and see how I felt. And if I was distressed, I could leave as opposed to while you’re here, you have to stay.”*

*FG 8*

*“I think they’d need to have obviously, like, training, … they need to have… a specialty in sort of like mental health, or, like, a psychology background or something like that, or just like something more than just being a nurse.”*

*FG 6*

*“However, I also know that part of peer support too, right? Like, it’s an aspect of it, they, you know, they work together. Yeah. So encouraging a culture of peer support, where there’s like training and education and a way to access help if you need instead of just relying on [organization’s employee assistance program].”*

*FG 8*

#### 3.2.3. A Break in the Clouds: Hope for Health

Nurse participants found it difficult to envision a future in their career amid all the barriers they are currently facing; there was, however, an overall desire to have a healthy workforce. Despite the challenges faced within their profession, there were still elements that the nurses could see that instilled hope and improvement in their future. They acknowledged the existing strengths of the profession and current context and felt that positive change and improved outcomes for those experiencing challenges in the workforce was possible.

##### Instilling Hope: Strengths and Potential

Participants reported that nurses individually and collectively have several strengths. These relate to their education, experience, and shared identity. Participants identified themselves as compassionate nurses who value relationships, wanting the best for their patients and colleagues. It was also reported that informal, volunteer peer-to-peer support was initiated to help with the current work environment and struggles nurses were facing. Resilience was perceived by the nurses as an important component of health and wellbeing. Participants also noted that they learned to be powerful advocates for themselves, their colleagues, and patients. It was acknowledged that an RP already exists within their organization for the emergency medical staff, and that this could be appropriately adapted for use by nurses. Furthermore, they expressed there are external support systems, such as the nursing union, that could be a resource moving forward to advocate for and enable effective and sustained change.

*“I think by the nature of being an ICU nurse, we are really good at advocating and we are really good at being vocal.”*

*FG 5*

*“Okay, well, it’s a giant province wide organization that already has programs in place in other places, right. Like I think there’s a strength there knowing that there is success with EMS [Emergency Medical Services], they have a reintegration program. Right. Like I think they are aware that this helps and evidence within their organization is a strength right because it like, well, it can help other groups, why can’t it help us.”*

*FG 8*

##### Possibilities and Outcomes

Participants recognized several areas of change that could be facilitated by an RP. Reducing stigma, both within the self and the workplace, was thought to potentially have positive outcomes on the overall health of nurses and the organization. They indicated that the inclusion of information about setting boundaries, additional mental health topics, and self-care could assist with longevity in the profession, and improve overall health and wellbeing.

*“If the organization were to implement something, where they do understand and empathize with what frontline nurses go through, then maybe staff would be more willing to work more often. It might help with staff morale….it might lead into other, you know, initiatives that can be established.”*

*FG 1*

They also recognized that an RP could benefit other health care professionals and hospital support staff, such as respiratory therapists, social workers, occupational therapists, doctors, and housekeeping.

*“Like respiratory therapists (RT), one of my good friends. He’s an RT and (during) COVID…he’s like, ‘I felt like I was just extubating one dead person after another for days on end.’ …social workers. Oh, my God, the things they do… I feel like social worker(s), RTs. And when I say nurses, it’s LPNs (licensed practical nurses). RNs (registered nurses), health care aides like it’s, it’s not just RNs? Yeah. The whole system. Everybody’s just struggled.”*

*FG 8*

Outside of the RP, participants suggested that adapting post-secondary education for nursing students would also be beneficial. 

### 3.3. Organizational Readiness for Implementation of Work Reintegration Initiatives

Organizational readiness for implementation of an RP among ER and ICU/CCU nurses was captured using the Hexagon Tool (Table 2). It is important to note that while the Hexagon Tool was used to explore readiness for implementation, it was not used to evaluate the RP itself. Need, Supports, Capacity, Fit, Usability, and Evidence were rated by the research team based on the collected data and current knowledge of the RP for PSP, as well as the state of the literature for workplace reintegration for nurses. Usability and Fit scores were the highest, while the perceived Need of the implementing site or organization for the RP was rated the lowest.

Recommendations for implementation of the RP follow based on program indicators of Need, Supports, Capacity, Fit, Usability, and Evidence.

#### 3.3.1. Need

The organization requires additional information regarding (1) issues facing nurses who are attempting to reintegrate into the workplace after mental health challenges, (2) the importance of a standardized approach to workplace reintegration, and (3) the RP itself. This could facilitate decision making around potential change to current return-to-work practices and utilization of the new program. It is recommended that this information be provided to the organization through advocacy efforts and further research. This may be initiated and facilitated by ongoing advocacy by healthcare professionals, unions, worker’s compensation organizations, researchers, and other stakeholders.

#### 3.3.2. Supports

Organizational support for nurses to attend RP facilitator training by allowing time away from the unit to attend would be facilitative. Attendance of the program by other key stakeholders, including decision- and policymakers, may also be beneficial for the purpose of information gathering on the potential Need, Fit, Usability, and Evidence regarding the RP. Further, implementation efforts would necessitate the allocation of resources to further assess fit in their context. It is recommended that the organization consider resourcing for exploration efforts, including putting policy, procedures, teams, RP facilitator training, and fidelity plans in place. Associated staffing, training, and further research would likely be required.

#### 3.3.3. Fit

The likely fit of the RP program within the organization’s current context was favorably assessed based on their values. Further intersectional factors and differences among the needs and roles of the diverse nursing staff have not yet been assessed. It is recommended that further consideration be given to the fit of the RP for nurses working outside of urban areas and on units beyond the ER or ICU/CCU, including inpatient, outpatient, and community-based settings.

#### 3.3.4. Usability

Monitoring the fidelity of implementation, scale, and spread is recommended for the use of the RP by both PSP and nursing populations. The collaborative co-design of a validated strategy by key stakeholders is advisable. These collaborators would include the implementing organization, the PSP organizations who have utilized and developed the program, and potentially other subject matter experts.

#### 3.3.5. Capacity

Allocation of financial and administrative support is recommended to further implement the RP with integrity. Doing so in the Exploration Phase of the potential implementation is recommended.

#### 3.3.6. Evidence

Further research is needed in several areas regarding the RP. This includes evaluating the efficacy of the RP and other peer-supported interventions among PSP [12,22,23]. There is also a lack of research regarding return-to-work and workplace reintegration of nurses, which needs to be further addressed. It is recommended that PSP and other organizations continue to collaborate with external research groups to facilitate high-quality research that can inform the future implementation, scale, and spread of the RP.

## 4. Discussion

This study aimed to explore the perceived need as well as the potential contextualization, adaption, and implementation of the RP for ER and ICU/CCU nurses through an implementation science lens. The mixed-methods approach, which combined qualitative analysis, questionnaire data, and an assessment of organizational readiness for implementation, provided results that were further triangulated. The findings may be utilized to inform further research, recommendations, and directions regarding workplace reintegration within a nursing population.

The participants in this study all identified as women, which reflects the women-dominated landscape of nurses in Canada, with 91% of the profession identifying as women [24]. Most of the participants were mid-career nurses. It was notable that all participants reported having been exposed to traumatic scenarios in the workplace, with a majority reporting distress due to these event(s) and seeking mental healthcare as a result. Less than half required time away from work; however, of those that did, only two were engaged in a return-to-work process.

A picture of the current setting participants faced in the ER and ICU/CCU, as well as their perceived needs, was painted through the thematic analysis. Challenges with return-to-work, nursing culture, stressed environments, and barriers to adoption of new innovations such as the RP were captured in the first theme. The perceived need for an RP in the current nursing context was outlined in the second theme, including the allotment of multiple resources and supports, education, and buy-in. These are consistent with findings from other PSP and key stakeholder studies that highlight the importance of these elements in the implementation and delivery of the RP [11,12]. Many participants were distrustful of key stakeholders, such as management, administration, and government, and felt it unlikely that change would be accepted and facilitated. The current work climate, noted barriers, and lack of response to their identified needs made trust difficult.

Although psychological distress due to workplace traumatic exposure was reported by most participants, it was notable from the questionnaire data that less than half of respondents sought mental health care. Engagement in some type of workplace reintegration or return-to-work process was reported only by a few participants who required time off work due to mental health challenges. This was consistent with the qualitative focus group data indicating that participants had to navigate return-to-work efforts on their own. Multiple reasons for the lack of engagement in organized workplace reintegration were cited by focus group participants, including stigma from oneself and peers, and an already stressed organization and environment that was unable to provide a program or assistance. Additionally, a lack of presumptive legislation [25] in the jurisdiction as it applies to nurses was seen as a reason that access to these programs was limited. Such a lack negates the mental health challenges that can result from cumulative exposure to trauma as opposed to a single index trauma. Placing the burden of proof on nurses was identified as a factor impeding their ability to access services for workplace reintegration. 

Amidst challenges envisioning change, some participants were able to identify the strengths of the profession, potential for improvement of the current state, and aspirations for the future. Their hope for a future state that was inclusive of workplace reintegration for multiple healthcare professions affected by mental health challenges was clear considering the potential for exposure to traumatic events among all staff working in their specific contexts. The overarching commitment of nurses to the health and wellbeing of all is exemplified by one participant comment:

*“Everyone wants everybody to succeed. I think at baseline, nurses want everyone to be happy, healthy, and safe in a personal and a professional way.”*

Envisioning how and if the RP could be contextualized or adapted for their population was challenging for participants, despite their agreement that a work RP for nurses was needed. Participants widely agreed that nurse-specific workplace reintegration policies, procedures, plans, and programs would assist those who have been off work due to mental health challenges. They felt that a sustainable, usable, context-specific, and evidence-based program adapted from the current PSP RP would have utility in this regard. Further exploration is yet to be carried out to gain a comprehensive understanding of the contextualization and potential adaptation required for this population.

Organizational readiness for the implementation of an RP among ER and ICU/CCU nurses in their current context was also explored, and recommendations identified based on program indicators of Need, Support, Capacity, Fit, Usability, and Evidence. Prior to initial implementation, the organization must further define *Need* through acquiring additional information regarding the challenges facing nurses, standardized approaches to return-to-work after an OSI, and the RP. Advocacy, further research, and engagement with staff and other stakeholders with lived experience in the ER and ICU/CCU would offer critical information. The organization would also need specific resources, including policies, procedures, teams, RP facilitator training, and fidelity plans, to be in place. Allocation of financial and administrative *Support* would be required, with consideration given to staffing. Determination of the *Fit* of the RP for nurses and potentially other healthcare professionals working outside of urban areas and on units beyond the ER, ICU/CCU would be essential for informing the potential contextualization and *Usability* of the RP in healthcare settings outside of acute care hospitals, including outpatient and community settings. Monitoring the fidelity of implementation, scale, and spread and gathering *Evidence* related to the program’s impact and effectiveness is recommended for both PSP and nursing populations if a pilot implementation of the RP were to be initiated. It would also be advantageous for fidelity plans to be collaboratively co-designed among different stakeholders of the RP. Finally, as there is a paucity of literature exploring workplace reintegration of nurses, further research efforts are imperative. It is recommended the organization and stakeholders of the RP work collaboratively with external researchers to further evaluate and inform approaches to workplace reintegration in the future. Overall, it is recommended that a structured approach to implementation and evaluation of future innovations such as an RP for nurses be guided by an evidence-based framework or model such as the AIFs.

Systemic and contextual factors outside the context of the RP cannot be ignored, as these were of the utmost importance to the study participants. Efforts to address the stressful healthcare environment, notably challenges around staffing, issues within nursing culture, and both self and societal stigma regarding mental health among nurses, are critically needed. Focused strategies to overcome such challenges might both reduce the potential for harm to nurses and increase the likelihood of success of an RP, were it to be implemented. Addressing such factors in a timely manner, however, will fundamentally require organizational and political will, intention, and resources, as well as additional research, advocacy, and innovation.

### Limitations of Study

There were a few limitations to this study. Despite widespread recruitment, participation was lower than expected. This was due, in part, to the current demands on the nursing participants. It was common for focus groups to be rescheduled multiple times or canceled completely in attempts to accommodate shift changes, overtime, and other demands on the nurses. This resulted in a smaller than expected sample size. Gender bias is notable in this study. Although efforts to recruit all genders were made, nursing remains a profession of mostly women, and this was reflected in the data [24]. Videoconferencing and the focus group setting may also contribute to potential bias, with participants being more concerned about privacy and confidentiality in this setting. As this is a small sample specific to a jurisdiction and healthcare system, the results are not generalizable to other contexts. Additionally, the nurses were only provided with a brief explanation and video demonstration of the RP, which may have not provided a full understanding of the RP in question. While the research team aimed to capture perspectives regarding adaptation of the RP for the nurses, this was challenging to isolate from the participants, as the cited barriers and needs clouded their vision for the future. Lastly, organizational readiness for implementation of the RP among ER and ICU/CCU nurses was based on responses from a small sample of nurses coupled with the research team’s knowledge of the RP. This analysis is a limited and narrow representation of program indicators for the RP in this context. More research and consultation with other stakeholders would be required for a more robust implementation science-based analysis.

## 5. Conclusions

Healthcare professionals, including nurses, are under unprecedented pressure in a strained healthcare climate. In the course of service, they are exposed to potentially traumatic, unpredictable, and morally ambiguous experiences that can result in OSIs. A lack of attention to the “reintegration” phase of mental health treatment for OSIs has created a functional gap for many individuals who are returning to the workplace after a psychological injury. As part of a holistic recovery model, initiatives are needed to treat OSIs and facilitate work reintegration processes after clinical intervention has concluded. There is demand among OSI-affected nurses for a more standardized, peer-supported workplace reintegration approach to support work reintegration. Exploration of innovative programs such as the RP created by PSP may address this need and provide additional support to nurses affected by mental health challenges. Research in this regard, however, is severely lacking and desperately needed. Investigation and evaluation of the RP through an implementation science lens would also be beneficial in the context of nurses. As the importance of effective workplace reintegration strategies becomes more recognized for certain professions, such as PSP and nurses, other professions may follow. Looking forward, effective, and evidence-based innovations and initiatives that increase the likelihood of a sustained return to work after injury could contribute to a stronger overall workforce. Not only do the health and wellbeing of our healthcare providers, including nurses and PSP, stand to benefit, but so too do the health and wellbeing of our communities, which are so integrally dependent on those who tirelessly serve the public.

## Figures and Tables

**Table 1:** Results of the participant questionnaire (*N* = 19).

Participant Demographics		Frequency (n/%)
Sex	Female	19/100
	Male	0/0
	Intersex	0/0
	Prefer not to say	0/0
Gender	Woman or feminine	19/100
	Man or masculine	0/0
	Transgender man, male, or masculine	0/0
	Transgender woman, female, or feminine	0/0
	Gender nonconforming, genderqueer, or gender-questioning	0/0
	Two-spirit	0/0
	Prefer not to specify	0/0
Ethnicity	White	18/95
	South Asian	1/5
	Chinese	0/0
	Black	0/0
	Filipino	0/0
	Latin American	0/0
	Arab	0/0
	Southeast Asian	0/0
	Korean	0/0
	Japanese	0/0
	Indigenous, Metis, Inuit	0/0
	Other/Unknown	0/0
	Prefer not to say	0/0
Professional Role	Registered Nurse	19/100
	Registered Psychiatric Nurse	0/0
	Licensed Practical Nurse	0/0
	Nurse Practitioner	0/0
Highest Level of Education	High school diploma	0/0
	Vocational or technical college	0/0
	College diploma	0/0
	Some undergraduate	0/0
	Undergraduate degree	17/89
	Graduate degree	2/11
Years in Profession	0–4	2/11
	5–9	3/15
	10–14	7/37
	15–19	6/32
	20–24	0/0
	25–29	1/5
	30+	0/0
Years within Provincial Health Organization	0–4	2/11
	5–9	6/32
	10–14	3/43
	15–19	8/42
	20–24	0/0
	25–29	0/0
	30+	0/0
Psychological Exposure to Trauma	Yes	19/100
	No	0/0
	No Answer/Prefer not to say	0/0
Psychological Distress as a Result of Trauma Exposure in the Workplace	Yes	17/89
	No	1/5
	No Answer/Prefer not to say	1/5
Sought Mental Health Care as a Result of Trauma Exposure in the Workplace	Yes	12/63
	No	6/32
	No Answer	1/5
Time off Work Due to Exposure to Psychological Trauma in the Workplace	Yes	8/42
	No	7/37
	Not Applicable	3/16
	No Answer/Prefer not to say	1/5
Participation in a Workplace Reintegration or Return-to-Work Process	Yes	2/11
	No	17/89
	No Answer/Prefer not to say	0/0

**Table 2:** Organizational readiness for implementation of work reintegration initiatives: the Hexagon Tool.

Program Indicator	Score (1 to 5)	Definition of Score	Rationale
Need	1	The implementing site has not demonstrated an understanding of how the program or practice meets the needs of the focus population.	The organization requires additional information on the RP and the potential need for workplace reintegration interventions within the profession of nursing to facilitate further understanding of the current issues and potential need.
Supports	2	Limited resources are available beyond a curriculum or one-time training.	Although the RP has a curriculum and facilitator training program, its accessibility to the nursing population is limited due to a lack of recognition of need by the implementing site. At this time, there are few resources available/allocated within the context of the implementing organization that would support implementation of the RP.
Capacity	2	The implementing site adopting the program or practice has the minimal capacity necessary, including only one of the following: a qualified workforce, financial supports, technology supports or administrative supports required to implement and sustain the program or practice with integrity.	Nurses are qualified to administer peer support for colleagues with OSI; however, financial supports, technology supports or administrative supports are not currently in place to implement and sustain the program or practice with integrity. There may be potential for this in the future.
Usability	4	The program or practice has operationalized principles and core components that are measurable and observable and has tools and resources to monitor fidelity, but does not have a validated fidelity measure; modifiable components are identified to support contextualization for new settings or focus populations.	The RP does have core components that can be measured both quantitatively and qualitatively. Examples of core components include exposure activities and drills and peer support practices, of which program evaluation techniques such as surveys and interviews can be used to measure effectiveness.There is not a defined and validated strategy for monitoring the fidelity of implementation, scale, and spread. Modifiable components are identified within the training curriculum for the RP to support contextualization for new settings or focus populations, such as nurses in different healthcare settings.
Fit	4	The program or practice fits with all of the priorities of the implementing site and community values; however, the values of racially, ethnically, culturally and linguistically specific populations and alignment with other initiatives have not been assessed for fit.	The organization emphasizes employee wellness and staff retention. It has a number of procedures, policies, and resources to improve these factors. The RP would fit the priorities of the implementing sites and community values, but intersectional factors and differences amongst needs and roles of the nursing staff have not been assessed for fit.
Evidence	2	The program or practice is guided by a well-developed theory of change or logic model for the focus population, and has demonstrated a relationship between the program or practice and outcomes based on an evaluation or practice-based evidence.	The RP is guided by a theory of change and has minimal peer-reviewed literature regarding PSP populations that involve evaluation. There is very limited literature regarding nursing and workplace reintegration. There is little literature concerning this RP and a nursing population.

## Data Availability

Data may be available upon request directly from the authors. Availability will be decided upon on a case-by-case basis at the author’s discretion in order to maximize the anonymity and confidentiality of the participants in this study.
